# Bis[4-(2-benzyl­idenepropyl­idene­amino)phen­yl] ether

**DOI:** 10.1107/S1600536809005005

**Published:** 2009-02-18

**Authors:** Mansoor Movahedi, Hassan Hadadzadeh, Karla Fejfarova, Michal Dusek, Aliakbar Dehno Khalaji

**Affiliations:** a Department of Science, Golestan University, Gorgan, Iran; b Department of Chemistry, Isfahan University of Technology, Isfahan 84156-83111, Iran; c Institute of Physics of the ASCR, Na Slovance 2, 182 21 Prague 8, Czech Republic; d Department of Science, Gorgan University of Agricultural Sciences and Natural Resources, Gorgan 49189-43464, Iran

## Abstract

The title compound, C_32_H_28_N_2_O, is a flexible Schiff base displaying a *trans* configuration across the C=N double bond. It was prepared in high yield by condensation of α-methyl­cinnamaldehyde and bis­(4-amino­phen­yl) ether in methanol at room temperature. The sample, with pseudo-ortho­rhom­bic cell parameters, exhibited merohedral twinning by rotation 180° around a*, with a refined twin domain fraction of 0.722 (1). The elongated shape of the elementary cell corresponds to the shape and direction of the mol­ecules. The dihedral angle between the O-linked aromatic rings is 57.86 (8)°.

## Related literature

For the synthesis of the title compound, see: Khalaji & Ng (2008 ▶). For related structures, see: Hu *et al.* (2008 ▶); Xu *et al.* (2008 ▶). For background to transition metal complexes, see: Laye (2007 ▶); Huang *et al.* (2005 ▶); Chu & Huang (2007 ▶).
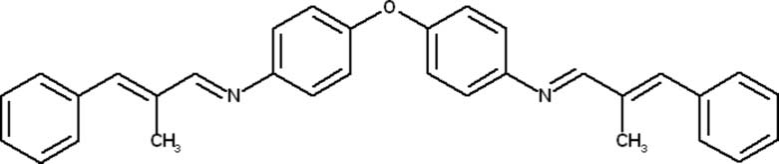


## Experimental

### 

#### Crystal data

C_32_H_28_N_2_O
*M* *_r_* = 456.6
Monoclinic, 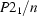

*a* = 7.4737 (3) Å
*b* = 55.929 (3) Å
*c* = 6.0275 (3) Å
β = 90.022 (4)°
*V* = 2519.5 (2) Å^3^
*Z* = 4
Cu *K*α radiation
μ = 0.56 mm^−1^
*T* = 295 K
0.51 × 0.38 × 0.02 mm

#### Data collection

Oxford Diffraction Gemini diffractometer with Atlas CCD detector
Absorption correction: multi-scan (*CrysAlis RED*; Oxford Diffraction, 2008 ▶) *T*_min_ = 0.682, *T*_max_ = 0.99
15440 measured reflections
3838 independent reflections
3428 reflections with *I* > 3σ(*I*)
*R*_int_ = 0.016
θ_max_ = 61.4°

#### Refinement

*R*[*F* ^2^ > 2σ(*F* ^2^)] = 0.036
*wR*(*F* ^2^) = 0.120
*S* = 2.32
3838 reflections
317 parameters
H-atom parameters constrained
Δρ_max_ = 0.19 e Å^−3^
Δρ_min_ = −0.18 e Å^−3^

### 

Data collection: *CrysAlis CCD* (Oxford Diffraction, 2008 ▶); cell refinement: *CrysAlis RED* (Oxford Diffraction, 2008 ▶); data reduction: *CrysAlis RED*; program(s) used to solve structure: *SIR2002* (Burla *et al.*, 2003 ▶); program(s) used to refine structure: *JANA2006* (Petříček *et al.*, 2008 ▶); molecular graphics: *DIAMOND* (Brandenburg & Putz, 2005 ▶); software used to prepare material for publication: *JANA2006*.

## Supplementary Material

Crystal structure: contains datablocks global, I. DOI: 10.1107/S1600536809005005/bt2870sup1.cif

Structure factors: contains datablocks I. DOI: 10.1107/S1600536809005005/bt2870Isup2.hkl

Additional supplementary materials:  crystallographic information; 3D view; checkCIF report

## Figures and Tables

**Fig. 1.**
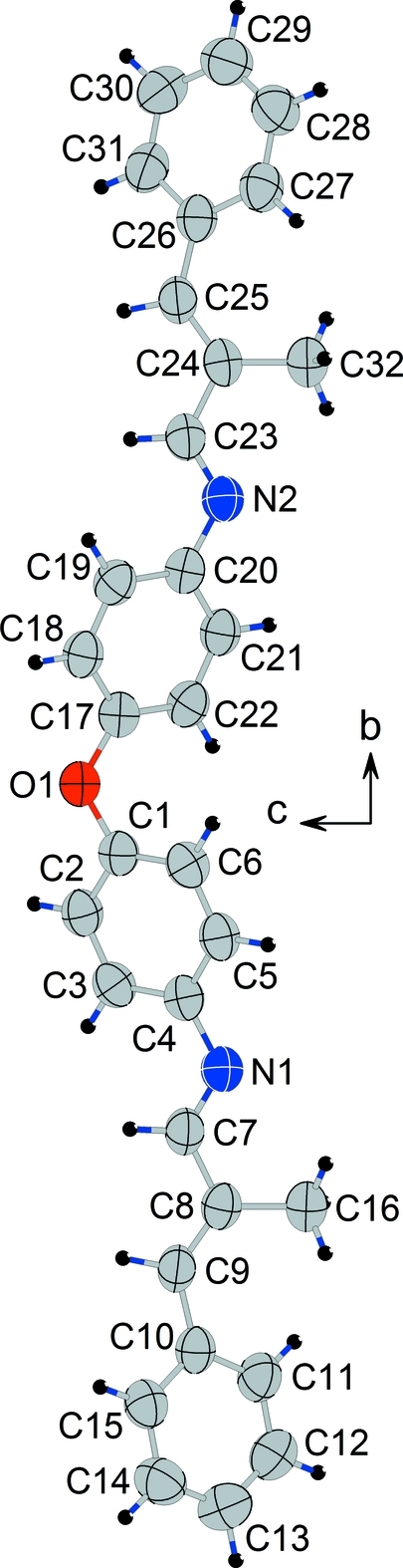
The molecule of (I) viewed along a with atom-labeling scheme. Displacement ellipsoids are drawn at the 50% probability level.

**Fig. 2.**
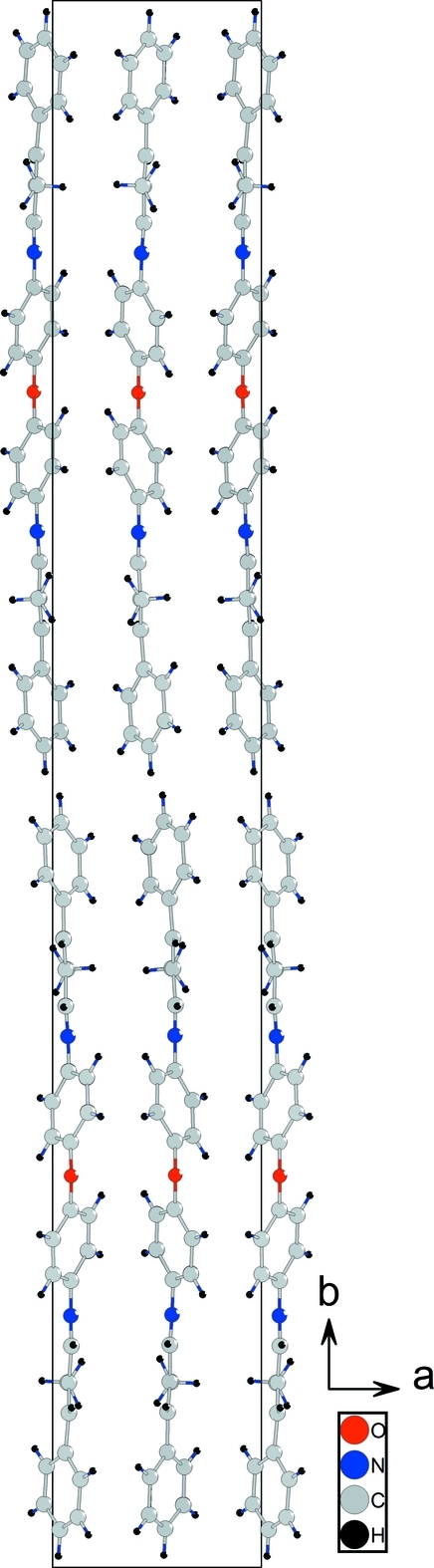
The packing of (I) viewed along c.

## supplementary crystallographic information

### Comment

Flexible Schiff-base ligands have received a lot of attention in the field of supramolecular coordination chemistry (Laye, 2007; Huang *et al.*, 2005; Chu & Huang, 2007). Because of easy syntheses of these compounds by condensation between aldehydes (or ketones) and amines, many of them were designed and sued to prepare transition metal complexes in recent years. Here, we report the synthesis and crystal structure of a new flexible Schiff-base compound (I). The molecule of (I) is shown in Fig. 1. Bond lengths and angles are comparable with those observed in similar compounds (Hu *et al.*, 2008; Xu *et al.*, 2008). The C(7)=N(1) and C(23)=N(2) bond lengths of 1.266 (3) and 1.270 (3) Å, respectively, conform to the usual value for a C=N double bond. Each half of the molecule displays a *trans* configuration across the C=N double bond. The prolongated shape of the molecule is reflected by very long *b* axis about 55 Å. The molecules are isolated (Fig. 2), with no intermolecular contacts. From crystallographic point of view merohedric twinning by rotation 180° around a* and pseudo-orthorhombic cell parameters should be noted. The twinning has occurred regularly in all tested samples.

### Experimental

The title compound  was synthesized using a method analogous to the literature procedure of Khalaji and Ng (2008). Crystals appropriate for data collection were obtained by slow evaporation from a methanol-chloroform mixture (1:5 *v*/*v*) at room temperature (yield; 88%).

### Refinement

All H atoms were found in difference Fourier maps, but they were constrained to ideal positions. The isotropic atomic displacement parameters of hydrogen atoms were set to 1.2*U*_eq_ of the parent atom. The sample had a pseudo-orthorhombic cell parameters and exhibited a merohedric twinning by rotation 180° around a* (with twinning matrix 1 0 0 / 0 - 1 0 / 0 0 - 1 applied to indices expressed like row vectors). The volume fraction of the second domain was refined to 0.278 (1).

### Crystal data

**Table:**

C_32_H_28_N_2_O	*F*(000) = 968
*M**_r_* = 456.6	*D*_x_ = 1.203 Mg m^−^^3^
Monoclinic, *P*2_1_/*n*	Cu *K*α radiation, λ = 1.54184 Å
Hall symbol: -P 2yn	Cell parameters from 9034 reflections
*a* = 7.4737 (3) Å	θ = 3.2–61.2°
*b* = 55.929 (3) Å	µ = 0.56 mm^−^^1^
*c* = 6.0275 (3) Å	*T* = 295 K
β = 90.022 (4)°	Plate, yellow
*V* = 2519.5 (2) Å^3^	0.51 × 0.38 × 0.02 mm
*Z* = 4	

### Data collection

**Table:**

Oxford Diffraction Gemini diffractometer with Atlas CCD detector	3838 independent reflections
Radiation source: X-ray tube	3428 reflections with *I* > 3σ(*I*)
mirrors	*R*_int_ = 0.016
Detector resolution: 20.7567 pixels mm^-1^	θ_max_ = 61.4°, θ_min_ = 3.2°
Rotation method data acquisition using ω scans	*h* = −8→8
Absorption correction: multi-scan (*CrysAlis RED*; Oxford Diffraction, 2008)	*k* = −62→62
*T*_min_ = 0.682, *T*_max_ = 0.99	*l* = −6→6
15440 measured reflections	

### Refinement

**Table:**

Refinement on *F*^2^	Primary atom site location: structure-invariant direct methods
Least-squares matrix: full	Secondary atom site location: difference Fourier map
*R*[*F*^2^ > 2σ(*F*^2^)] = 0.036	Hydrogen site location: difference Fourier map
*wR*(*F*^2^) = 0.120	H-atom parameters constrained
*S* = 2.32	Weighting scheme based on measured s.u.'s *w* = 1/[σ^2^(*I*) + 0.0016*I*^2^]
3838 reflections	(Δ/σ)_max_ = 0.001
317 parameters	Δρ_max_ = 0.19 e Å^−^^3^
0 restraints	Δρ_min_ = −0.18 e Å^−^^3^
112 constraints	

### Special details

**Table:**

Refinement. The refinement was carried out against all reflections. The conventional *R*-factor is always based on *F*. The goodness of fit as well as the weighted *R*-factor are based on *F* and *F*^2^ for refinement carried out on *F* and *F*^2^, respectively. The threshold expression is used only for calculating *R*-factors *etc*. and it is not relevant to the choice of reflections for refinement.The program used for refinement, Jana2006, uses the weighting scheme based on the experimental expectations, see _refine_ls_weighting_details, that does not force *S* to be one. Therefore the values of *S* are usually larger than the ones from the *SHELX* program.

### Fractional atomic coordinates and isotropic or equivalent isotropic displacement parameters (Å^2^)

**Table:**

	*x*	*y*	*z*	*U*_iso_*/*U*_eq_	
O1	0.41159 (19)	0.25020 (2)	1.1527 (2)	0.0638 (4)	
N1	0.4123 (2)	0.33936 (3)	0.7401 (3)	0.0617 (5)	
N2	0.4264 (2)	0.16126 (3)	0.7377 (3)	0.0623 (5)	
C1	0.4131 (2)	0.27183 (3)	1.0403 (3)	0.0548 (5)	
C2	0.4984 (2)	0.29047 (3)	1.1464 (3)	0.0613 (6)	
C3	0.4970 (2)	0.31299 (3)	1.0534 (3)	0.0635 (6)	
C4	0.4118 (2)	0.31709 (3)	0.8503 (3)	0.0568 (5)	
C5	0.3295 (2)	0.29795 (3)	0.7467 (3)	0.0601 (6)	
C6	0.3268 (2)	0.27553 (3)	0.8402 (3)	0.0616 (6)	
C7	0.4096 (2)	0.35875 (3)	0.8483 (3)	0.0636 (6)	
C8	0.4215 (2)	0.38208 (3)	0.7420 (3)	0.0600 (6)	
C9	0.4227 (2)	0.40156 (3)	0.8727 (3)	0.0639 (6)	
C10	0.4388 (2)	0.42689 (3)	0.8176 (3)	0.0602 (6)	
C11	0.5287 (3)	0.43555 (4)	0.6318 (4)	0.0723 (7)	
C12	0.5396 (3)	0.45964 (4)	0.5896 (4)	0.0859 (9)	
C13	0.4641 (3)	0.47591 (4)	0.7318 (4)	0.0875 (9)	
C14	0.3778 (3)	0.46796 (4)	0.9185 (4)	0.0842 (8)	
C15	0.3661 (3)	0.44386 (4)	0.9620 (4)	0.0712 (7)	
C16	0.4273 (3)	0.38214 (4)	0.4939 (3)	0.0780 (8)	
C17	0.4135 (2)	0.22865 (3)	1.0395 (3)	0.0555 (5)	
C18	0.3308 (2)	0.20971 (3)	1.1439 (3)	0.0613 (6)	
C19	0.3356 (2)	0.18727 (3)	1.0517 (3)	0.0628 (6)	
C20	0.4223 (2)	0.18353 (3)	0.8484 (3)	0.0574 (5)	
C21	0.5012 (2)	0.20285 (3)	0.7441 (3)	0.0606 (6)	
C22	0.5005 (2)	0.22525 (3)	0.8389 (3)	0.0612 (6)	
C23	0.4375 (2)	0.14192 (4)	0.8456 (3)	0.0648 (6)	
C24	0.4369 (2)	0.11855 (3)	0.7384 (3)	0.0609 (6)	
C25	0.4481 (2)	0.09907 (3)	0.8688 (3)	0.0644 (6)	
C26	0.4490 (2)	0.07366 (3)	0.8136 (3)	0.0612 (6)	
C27	0.3678 (3)	0.06393 (4)	0.6255 (4)	0.0716 (7)	
C28	0.3726 (3)	0.03972 (4)	0.5842 (4)	0.0843 (8)	
C29	0.4589 (3)	0.02459 (4)	0.7274 (4)	0.0854 (8)	
C30	0.5381 (3)	0.03360 (4)	0.9145 (4)	0.0862 (9)	
C31	0.5324 (3)	0.05778 (4)	0.9586 (4)	0.0741 (7)	
C32	0.4306 (3)	0.11845 (4)	0.4907 (3)	0.0773 (8)	
H2	0.558692	0.287765	1.284787	0.0735*	
H3	0.555012	0.325979	1.128755	0.0762*	
H5	0.272697	0.300391	0.605623	0.0721*	
H6	0.265779	0.262608	0.76752	0.0739*	
H11	0.584073	0.424477	0.531363	0.0868*	
H12	0.600738	0.46515	0.459163	0.1031*	
H13	0.471712	0.492723	0.700898	0.105*	
H14	0.325198	0.479257	1.01914	0.1011*	
H15	0.306821	0.438587	1.094548	0.0855*	
H18	0.26953	0.212226	1.281899	0.0736*	
H19	0.279415	0.174115	1.126614	0.0753*	
H21	0.557768	0.200601	0.602612	0.0728*	
H22	0.559553	0.238363	0.76685	0.0734*	
H27	0.307393	0.074305	0.523036	0.0859*	
H28	0.315201	0.033414	0.454239	0.1011*	
H29	0.463802	0.00776	0.696717	0.1024*	
H30	0.597998	0.023017	1.01551	0.1035*	
H31	0.586783	0.063783	1.091686	0.0889*	
H7	0.399203	0.358045	1.006914	0.0763*	
H9	0.410947	0.398259	1.028338	0.0767*	
H23	0.446597	0.142663	1.004279	0.0778*	
H25	0.457048	0.102463	1.024492	0.0773*	
H16a	0.359347	0.36888	0.438036	0.0936*	
H16b	0.37714	0.396808	0.439336	0.0936*	
H16c	0.549168	0.380782	0.445248	0.0936*	
H32a	0.493257	0.132162	0.434681	0.0927*	
H32b	0.486302	0.104148	0.435939	0.0927*	
H32c	0.308313	0.118963	0.442292	0.0927*	

### Atomic displacement parameters (Å^2^)

**Table:**

	*U*^11^	*U*^22^	*U*^33^	*U*^12^	*U*^13^	*U*^23^
O1	0.0715 (8)	0.0641 (7)	0.0559 (7)	−0.0001 (5)	0.0032 (7)	0.0004 (6)
N1	0.0581 (8)	0.0672 (9)	0.0598 (9)	−0.0006 (6)	0.0011 (7)	0.0000 (7)
N2	0.0611 (8)	0.0675 (9)	0.0585 (9)	−0.0007 (7)	0.0045 (7)	0.0004 (7)
C1	0.0445 (8)	0.0641 (10)	0.0559 (9)	0.0018 (7)	0.0061 (7)	−0.0017 (8)
C2	0.0571 (9)	0.0713 (11)	0.0554 (9)	0.0006 (8)	−0.0064 (8)	−0.0025 (9)
C3	0.0600 (10)	0.0670 (11)	0.0634 (11)	−0.0034 (8)	−0.0054 (8)	−0.0080 (9)
C4	0.0503 (8)	0.0659 (10)	0.0543 (9)	0.0003 (7)	0.0039 (8)	−0.0023 (8)
C5	0.0541 (9)	0.0719 (11)	0.0543 (9)	0.0012 (7)	−0.0042 (8)	−0.0032 (8)
C6	0.0505 (8)	0.0688 (11)	0.0655 (11)	−0.0027 (7)	−0.0066 (8)	−0.0065 (9)
C7	0.0698 (11)	0.0701 (11)	0.0508 (9)	0.0010 (8)	0.0035 (9)	0.0014 (9)
C8	0.0553 (9)	0.0703 (11)	0.0543 (10)	0.0018 (8)	0.0024 (8)	0.0043 (8)
C9	0.0703 (11)	0.0720 (11)	0.0495 (9)	0.0028 (8)	0.0028 (8)	0.0042 (9)
C10	0.0555 (9)	0.0704 (11)	0.0547 (10)	0.0015 (8)	−0.0040 (8)	0.0020 (8)
C11	0.0742 (11)	0.0766 (13)	0.0662 (11)	0.0011 (9)	0.0119 (10)	0.0048 (10)
C12	0.0964 (15)	0.0807 (14)	0.0807 (15)	−0.0061 (11)	0.0132 (12)	0.0151 (11)
C13	0.0925 (15)	0.0701 (13)	0.1000 (17)	−0.0013 (11)	−0.0004 (14)	0.0106 (12)
C14	0.0870 (14)	0.0745 (13)	0.0912 (16)	0.0068 (10)	0.0064 (13)	−0.0108 (12)
C15	0.0702 (12)	0.0789 (13)	0.0645 (11)	−0.0009 (9)	0.0054 (9)	−0.0040 (9)
C16	0.0973 (16)	0.0812 (13)	0.0555 (11)	−0.0032 (11)	0.0008 (11)	0.0019 (9)
C17	0.0456 (8)	0.0639 (10)	0.0572 (9)	0.0009 (7)	−0.0010 (7)	0.0014 (8)
C18	0.0559 (9)	0.0714 (11)	0.0568 (10)	0.0012 (8)	0.0096 (8)	0.0040 (9)
C19	0.0580 (9)	0.0670 (11)	0.0634 (11)	−0.0028 (8)	0.0117 (8)	0.0062 (9)
C20	0.0493 (8)	0.0668 (10)	0.0562 (9)	0.0013 (7)	0.0027 (8)	0.0026 (8)
C21	0.0560 (9)	0.0718 (11)	0.0541 (9)	0.0001 (7)	0.0104 (8)	0.0029 (8)
C22	0.0539 (9)	0.0674 (11)	0.0623 (10)	−0.0032 (7)	0.0109 (8)	0.0061 (8)
C23	0.0715 (11)	0.0708 (11)	0.0522 (9)	0.0025 (8)	0.0015 (9)	−0.0015 (9)
C24	0.0593 (10)	0.0709 (11)	0.0524 (10)	0.0004 (8)	0.0033 (8)	−0.0021 (8)
C25	0.0706 (11)	0.0733 (11)	0.0493 (9)	−0.0008 (8)	0.0002 (8)	−0.0044 (9)
C26	0.0570 (9)	0.0723 (11)	0.0543 (10)	−0.0016 (8)	0.0022 (8)	−0.0002 (8)
C27	0.0707 (11)	0.0774 (12)	0.0667 (12)	−0.0016 (9)	−0.0099 (10)	−0.0022 (10)
C28	0.0925 (15)	0.0827 (14)	0.0776 (14)	−0.0149 (11)	−0.0013 (12)	−0.0131 (12)
C29	0.0934 (15)	0.0725 (13)	0.0901 (16)	−0.0043 (11)	0.0137 (13)	−0.0041 (12)
C30	0.0947 (15)	0.0747 (13)	0.0892 (16)	0.0011 (11)	−0.0021 (13)	0.0167 (12)
C31	0.0793 (13)	0.0799 (14)	0.0630 (11)	−0.0062 (10)	−0.0057 (10)	0.0078 (10)
C32	0.0954 (15)	0.0798 (13)	0.0567 (11)	−0.0036 (11)	0.0032 (11)	−0.0016 (9)

### Geometric parameters (Å, °)

**Table:**

O1—C1	1.386 (2)	C16—H16a	0.96
O1—C17	1.385 (2)	C16—H16b	0.96
N1—C4	1.412 (2)	C16—H16c	0.96
N1—C7	1.266 (2)	C17—C18	1.378 (2)
N2—C20	1.414 (2)	C17—C22	1.386 (3)
N2—C23	1.264 (2)	C18—C19	1.373 (3)
C1—C2	1.379 (2)	C18—H18	0.96
C1—C6	1.384 (3)	C19—C20	1.402 (3)
C2—C3	1.379 (3)	C19—H19	0.96
C2—H2	0.96	C20—C21	1.383 (3)
C3—C4	1.398 (3)	C21—C22	1.377 (3)
C3—H3	0.96	C21—H21	0.96
C4—C5	1.384 (2)	C22—H22	0.96
C5—C6	1.375 (3)	C23—C24	1.458 (3)
C5—H5	0.96	C23—H23	0.96
C6—H6	0.96	C24—C25	1.346 (3)
C7—C8	1.456 (3)	C24—C32	1.494 (3)
C7—H7	0.96	C25—C26	1.459 (3)
C8—C9	1.345 (3)	C25—H25	0.96
C8—C16	1.496 (3)	C26—C27	1.396 (3)
C9—C10	1.460 (3)	C26—C31	1.393 (3)
C9—H9	0.96	C27—C28	1.377 (3)
C10—C11	1.393 (3)	C27—H27	0.96
C10—C15	1.398 (3)	C28—C29	1.370 (3)
C11—C12	1.374 (3)	C28—H28	0.96
C11—H11	0.96	C29—C30	1.370 (4)
C12—C13	1.372 (3)	C29—H29	0.96
C12—H12	0.96	C30—C31	1.379 (3)
C13—C14	1.372 (4)	C30—H30	0.96
C13—H13	0.96	C31—H31	0.96
C14—C15	1.376 (3)	C32—H32a	0.96
C14—H14	0.96	C32—H32b	0.96
C15—H15	0.96	C32—H32c	0.96
			
C1—O1—C17	121.25 (14)	H16b—C16—H16c	109.4707
C4—N1—C7	120.89 (16)	O1—C17—C18	116.07 (16)
C20—N2—C23	120.83 (16)	O1—C17—C22	123.64 (15)
O1—C1—C2	115.92 (15)	C18—C17—C22	120.20 (16)
O1—C1—C6	123.52 (15)	C17—C18—C19	120.40 (17)
C2—C1—C6	120.45 (16)	C17—C18—H18	119.7986
C1—C2—C3	119.92 (17)	C19—C18—H18	119.8003
C1—C2—H2	120.0388	C18—C19—C20	120.14 (16)
C3—C2—H2	120.0374	C18—C19—H19	119.9296
C2—C3—C4	120.58 (17)	C20—C19—H19	119.9325
C2—C3—H3	119.7093	N2—C20—C19	123.66 (15)
C4—C3—H3	119.7097	N2—C20—C21	117.67 (16)
N1—C4—C3	123.73 (15)	C19—C20—C21	118.60 (16)
N1—C4—C5	118.11 (16)	C20—C21—C22	121.35 (17)
C3—C4—C5	118.09 (16)	C20—C21—H21	119.326
C4—C5—C6	121.81 (17)	C22—C21—H21	119.3271
C4—C5—H5	119.0935	C17—C22—C21	119.28 (16)
C6—C5—H5	119.0919	C17—C22—H22	120.3631
C1—C6—C5	119.12 (16)	C20—C22—H22	149.7198
C1—C6—H6	120.4391	N2—C23—C24	122.61 (18)
C5—C6—H6	120.4404	N2—C23—H23	118.6934
N1—C7—C8	122.68 (17)	C24—C23—H23	118.6931
N1—C7—H7	118.6563	C23—C24—C25	117.85 (17)
C8—C7—H7	118.6616	C23—C24—C32	116.50 (16)
C7—C8—C9	117.94 (17)	C25—C24—C32	125.61 (17)
C7—C8—C16	116.35 (16)	C24—C25—C26	130.95 (17)
C9—C8—C16	125.70 (17)	C24—C25—H25	114.5252
C8—C9—C10	130.80 (17)	C26—C25—H25	114.5237
C8—C9—H9	114.598	C25—C26—C27	124.25 (17)
C10—C9—H9	114.6038	C25—C26—C31	118.67 (17)
C9—C10—C11	124.07 (17)	C27—C26—C31	117.06 (18)
C9—C10—C15	119.02 (17)	C26—C27—C28	121.3 (2)
C11—C10—C15	116.87 (18)	C26—C27—H27	119.375
C10—C11—C12	121.3 (2)	C28—C27—H27	119.3729
C10—C11—H11	119.3764	C27—C28—C29	120.4 (2)
C12—C11—H11	119.3736	C27—C28—H28	119.8062
C11—C12—C13	120.7 (2)	C29—C28—H28	119.8072
C11—C12—H12	119.6501	C28—C29—C30	119.7 (2)
C13—C12—H12	119.6503	C28—C29—H29	120.1738
C12—C13—C14	119.4 (2)	C30—C29—H29	120.1722
C12—C13—H13	120.2841	C29—C30—C31	120.4 (2)
C14—C13—H13	120.2831	C29—C30—H30	119.7998
C13—C14—C15	120.3 (2)	C31—C30—H30	119.7949
C13—C14—H14	119.8703	C26—C31—C30	121.2 (2)
C15—C14—H14	119.8702	C26—C31—H31	119.3916
C10—C15—C14	121.5 (2)	C30—C31—H31	119.3905
C10—C15—H15	119.2735	C24—C32—H32a	109.4703
C14—C15—H15	119.2764	C24—C32—H32b	109.4721
C8—C16—H16a	109.4718	C24—C32—H32c	109.4709
C8—C16—H16b	109.4708	H32a—C32—H32b	109.4716
C8—C16—H16c	109.4712	H32a—C32—H32c	109.4711
H16a—C16—H16b	109.4705	H32b—C32—H32c	109.4713
H16a—C16—H16c	109.4723		
			
?—?—?—?	?		

### Hydrogen-bond geometry (Å, °)

**Table:**

*D*—H···*A*	*D*—H	H···*A*	*D*···*A*	*D*—H···*A*
?—?···?	?	?	?	?
